# Age-Stratified Analysis of COVID-19 Outcome Using Machine Learning Predictive Models

**DOI:** 10.3390/healthcare10102027

**Published:** 2022-10-14

**Authors:** Juan L. Domínguez-Olmedo, Álvaro Gragera-Martínez, Jacinto Mata, Victoria Pachón

**Affiliations:** 1 I2C Research Group, Higher Technical School of Engineering, University of Huelva, 21007 Huelva, Spain; 2 Research Center for Technology, Energy and Sustainability (CITES), University of Huelva, 21007 Huelva, Spain; 3 Juan Ramón Jiménez University Hospital, 21005 Huelva, Spain

**Keywords:** COVID-19, machine learning, prediction, feature importance

## Abstract

Since the emergence of COVID-19, most health systems around the world have experienced a series of spikes in the number of infected patients, leading to collapse of the health systems in many countries. The use of clinical laboratory tests can serve as a discriminatory method for disease severity, defining the profile of patients with a higher risk of mortality. In this paper, we study the results of applying predictive models to data regarding COVID-19 outcome, using three datasets after age stratification of patients. The extreme gradient boosting (XGBoost) algorithm was employed as the predictive method, yielding excellent results. The area under the receiving operator characteristic curve (AUROC) value was 0.97 for the subgroup of patients up to 65 years of age. In addition, SHAP (Shapley additive explanations) was used to analyze the feature importance in the resulting models.

## 1. Introduction

The beginning of the SARS-CoV-2 pandemic at the end of 2019 resulted in changes in people’s way of life, their relationships with others and changes in work, and above all it has posed and currently continues to constitute one of the greatest societal challenges of our time. In this sense, the health crisis is noteworthy, not only due to the mortality of the coronavirus, but also because of the indirect consequences it has generated, since many patients have been unable to be diagnosed and treated for other pathologies. We must also add the economic consequences that this pandemic is causing in every country [1].

The coronavirus occurs in many patients, initially presenting with respiratory disease, which in some cases later derives in respiratory failure and death in a significant percentage of those infected. Respiratory involvement is not the exclusive symptom of COVID-19, since many other patients develop a severe acute respiratory syndrome, which occurs with an increase in pro-inflammatory cytokines, which can cause systemic inflammation with multi-organ failure and death [2]. On the other hand, a significant percentage of patients develop coagulation alterations, entering a prothrombotic state; these patients undergo thrombi in the lungs, brain and elsewhere in the body, which on many occasions lead to the patient’s death [3].

Since the emergence of COVID-19, most health systems around the world have experienced a series of spikes in the number of infected patients. This exponential increase in cases has caused an unparalleled collapse of the health systems in many countries. Healthcare systems have come under pressure to such an extent that it has been necessary to choose which patients are candidates for treatment and which are not. In addition, in the initial moments of the pandemic rapid diagnostic methods were practically unavailable, leading to several patients not receiving adequate treatment [4].

To prioritize the treatment of infected patients, the World Health Organization (WHO), together with the USA’s Center for Disease Control and Prevention (CDC), defined the profile of patients with a higher risk of mortality. The risk group was defined as patients over 65 years old, those who lived in a nursing home and people with at least one of the following health problems: chronic lung disease, severe heart disease, obesity, diabetes, hypertension, kidney failure, liver disease or patients with immunodeficiency [5]. This classification of risk factors has evolved, making it necessary to stratify according to patient’s age.

Machine learning is a branch of artificial intelligence that allows machines to make systems capable of identifying patterns in data to make predictions. It has been successfully applied to tasks related to healthcare [6,7]. By applying machine learning techniques to clinical laboratory data from patients’ hospital stays [8], we developed mortality prediction models after stratification of patients according to age (up to 65 years, 65–80 years and older than 80 years). For each age group we also studied the influence of laboratory parameters on the model predictions. This enables healthcare professionals to provide more personalized attention to patients, focusing on possible complications depending on the age group, which translates into a better evolution of the disease in these patients.

The rest of this paper is organized as follows. The Section 2 gives a description of the data and methods employed. Section 3 and Section 4 detail and discuss the results obtained. Finally, some concluding remarks are stated in Section 5.

## 2. Materials and Methods

In this section, the dataset used in this work is described first. Next, a detailed description of the machine learning techniques employed is provided.

### 2.1. Materials

In this study, we used anonymized clinical data provided by a private Spanish hospital group (HM Hospitales), which has facilities mainly in the Autonomous Communities of Madrid and Galicia, and also in the city of Barcelona. This group made its data available to the scientific community for research purposes.

Using these electronic medical records, we used the available data of patients admitted with a possible diagnosis of COVID-19 between March and June 2020. From all the data tables provided, the following were selected:

- A main table containing specific data on patient hospitalization (2547 records).
- A table of laboratory data with the results of different tests performed on patients both on admission to the hospital and during their hospitalization (584,136 records).

In the main table there is an ‘Outcome’ feature, with the values: ‘Death’ (15.0%), ‘Home’ (77.2%), ‘Transfer to hospital’ (3.7%), ‘Transfer to socio-sanitary center’ (2.1%), ‘Voluntary discharge’ (0.1%) and without value (1.9%).

### 2.2. Methods

#### 2.2.1. Data Preprocessing

As an initial step in any machine learning procedure, a data preprocessing stage was performed. The information from the two tables was preprocessed as follows:

- Selection of patients with a confirmed diagnosis of SARS-CoV-2 with an ‘Outcome’ value of ‘Home’ or ‘Death’, to analyze the data likely related to the patient’s survival.
- Combination of data from both tables using the patient ID. Since patients may present a variable number of results for each laboratory parameter, the mean value was calculated and assigned to each of them.
- Due to the considerable number of missing values in the laboratory parameters, a filtering process was carried out both in the records and the features to obtain a clean dataset with no missing values. In this work, for the sake of uniformity and simplicity, we used the following procedure: first, we eliminated those features that had missing values in more than 10% of all records; subsequently, only those records with values in the set of features selected in the previous step were selected.
- The values of the discrete features were transformed into numerical values. Among all the features used in this study, the only two variables with discrete values were ‘Sex’ and ‘Outcome’, which are the classes to be predicted. Both features have two possible values, so they were transformed into binary values.

#### 2.2.2. XGBoost

The algorithm XGBoost is a gradient boosted decision tree model presented by Chen and Guestrin [9]. It offers parallel tree boosting to address a wide range of data science problems quickly and accurately. It is an open-source software library which provides a gradient boosting framework designed to be highly efficient and flexible. This algorithm stands out due to its ability to produce accurate predictions which are often comparable to or better than those produced by more computationally complex models.

This method trains decision trees sequentially and adds a new decision tree to the previous one if it improves the objective function’s value. During training, XGBoost produces several “weak” prediction models sequentially (decision trees), and then it uses the effects of the previous model to construct a “stronger” model with improved predictive strength and greater stability in its results.

This algorithm aims to minimize the objective functions by adjusting the parameters in each tree. An optimization algorithm is used to select which tree to add to create a stronger model. Each model is compared to the one before it. When a new model performs better, it is added to the strong model. If, on the other hand, the outcomes are poor, it is returned to the best previous one and updated in a different way.

This method is repeated until the gap between successive models is negligible, indicating that we have found the best possible model. XGBoost has been successfully applied to medicine tasks, such as prediction of diabetes risk [10], hypertension [11], drug response [12], smoking-induced diseases [13], lung cancer [14], mortality in elderly patients [15] or bone mass loss [16].

In this study, all experiments were performed using the XGBoost library for Python [17].

#### 2.2.3. Feature Importance

A crucial feature in machine learning research is the interpretability of the results. In the field of medicine, this characteristic is paramount for healthcare professionals to be able to draw conclusions and make decisions based on the results obtained using machine learning algorithms [18]. Machine learning interpretability can be defined as the degree to which the user can understand and interpret a prediction made by a model [19].

In this paper, we used the SHAP framework to assist in interpreting machine learning models [20]. SHAP is based on game theory [21] and enables the identification and ranking of the features that most influence the prediction model, selecting the optimal set from these features.

This technique can assign values to the importance of features in complex machine learning models, providing an interpretable prediction for a test sample. SHAP values have been proposed as a unified metric of feature significance, since they assign an importance value to each feature that reflects the impact of including that feature in the model prediction, and they can be calculated according to (1):

(1) $$\emptyset_{i}\left(v\right)=\overset{\;}{\underset{S\subseteq N\backslash \left\{i\right\}}{\sum }}\frac{\left|S\right|!\left(n-\left|S\right|-1\right)!}{n!}\left(v(S\cup^{\;}\left\{i\right\}\right)-v\left(S\right))$$

Using SHAP with XGBoost has an additional advantage, which is the possibility of using TreeSHAP, a quick variant of SHAP for tree-based machine learning. It enables the exact computation of Shapley values in polynomial time [22].

**Figure 1:**
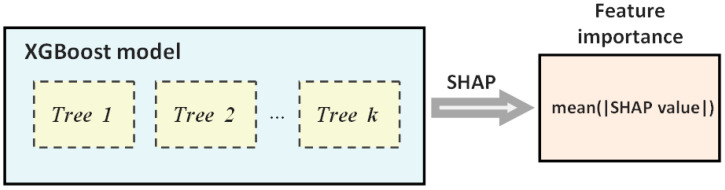
Diagram depicting the process for calculating feature importance according to SHAP values for each feature in each instance of the dataset.

Figure 1 outlines the procedure used in this work. The importance of a feature is calculated as the average of the absolute SHAP values for all the instances of the dataset.

#### 2.2.4. Feature Selection

In statistics and machine learning, feature selection involves the procedure to select the subset of relevant features (variables) and use it to build a model. Some possible advantages that can derive from a reduction in the features used include: a reduced processing time to train the model, a simplified model that is more interpretable and an improved generalization capability in the model.

It is important to consider that there may be some features that may be redundant or irrelevant, meaning that they can be omitted without affecting the quality of the model obtained [23].

In this work, a simple feature selection technique was chosen, which can be considered within the category of wrapper methods, with the aim of reducing the number of features used without affecting the performance of the model. Moreover, in the field of medicine, where this study is developed, considering a smaller number of features has an additional advantage in this case. Because laboratory test results are involved, the time and cost associated with analytical tests can be reduced, and medical professionals can focus their efforts on the characteristics that most influence the model’s output.

**Figure 2:**
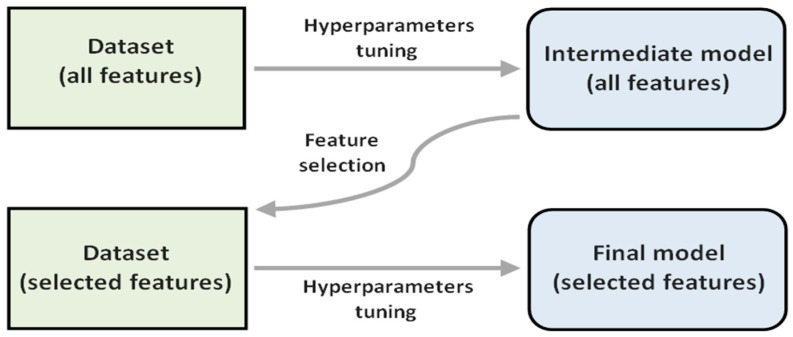
Process to obtain a final model after applying feature selection on an intermediate model.

The procedure involved uses the feature importance values, which in this work are related to the SHAP values, to select a subset of features. Specifically, we selected the 12 features with the highest feature importance value in a first model (intermediate model) after performing a hyperparameter tuning process. Then, using only these 12 features, the hyperparameter tuning process was repeated to obtain the final model. Figure 2 shows a diagram of the described procedure.

#### 2.2.5. Hyperparameter Optimization

During the training phase of a predictive model, it is generally necessary to fix the values of certain parameters (hyperparameters). Among the variety of existing parameters in XGBoost, some of the most relevant ones were considered for adjustment, leaving the rest at their default values. The six parameters selected for tuning affect both the number and structure of the gradient boosted trees (n_estimators, max_depth, and min_child_weight), as well as the learning process itself (learning_rate, subsample, and colsample_bytree).

The values for these hyperparameters were chosen using hyperopt, a Python library for distributed hyperparameter optimization [24]. The metric used in the optimization was the AUROC (area under the receiving operator characteristic curve), and the algorithm used was the tree-structured Parzen estimator (TPE). It was carried out in 1000 tuning cycles, and in each tuning cycle 10-fold stratified cross-validation was used to estimate the value of the AUROC.

#### 2.2.6. Evaluation Metrics

In order to evaluate the performance of the predictive models obtained, the AUROC value was calculated, based on its ROC (receiver operating characteristics) curve, which plots the true-positive rate against the false-positive rate. In addition, the accuracy value was calculated (2), and also, by determining the cut-point that maximizes the Youden index (3), the associated sensitivity (4) and specificity (5) values [25].

(2) $$accuracy=\frac{true\;positives+true\;negatives}{true\;positives+false\;positives+true\;negatives+false\;negatives}$$

(3) $$sensitivity=\frac{true\;positives}{true\;positives+false\;negatives}$$

(4) $$specificity=\frac{true\;negatives}{true\;negatives+false\;positives}$$

(5) J=sensitivity+specificity−1

The calculation of these metrics was carried out using the bootstrap method, which iteratively resamples a dataset with replacement [26], and goes through 200 iterations for this purpose.

## 3. Results

After the initial preprocessing phase, data from 1823 patients were selected. Given that this study focuses on comparing results according to age subgroups, the resulting dataset was divided according to the patients’ age. Thus, three datasets were obtained: the first with patients under 65 years of age, the second with those between 66 and 80 years of age and the third with those over 80 years of age.

Table 1 shows information about these datasets, detailing the distribution of patients according to ‘Sex’ and ‘Outcome’. The number of selected features was 32, corresponding to age, sex and laboratory test results. Table A1 in Appendix A provides the features corresponding to laboratory tests, together with their units and reference values.

### 3.1. Evaluation of the Models

**Figure 3:**
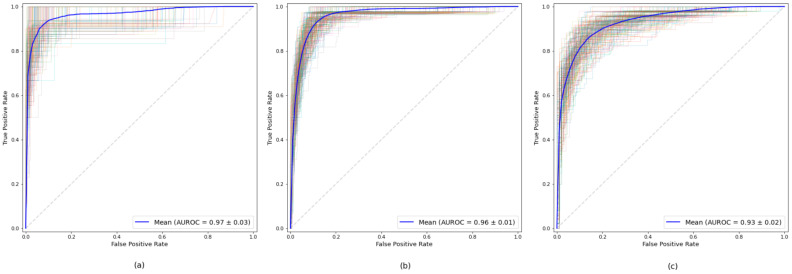
ROC curves for the final models for each age subgroup: (**a**) up to 65 years, (**b**) between 66 and 80, (**c**) older than 80 years. The curves for each bootstrap evaluation are shown, along with the average value (blue).

In order to evaluate the final models obtained, we used the bootstrap technique, calculating the average value for the different metrics described above. Table 2 shows the results obtained for each of the established age subgroup. For all three datasets, high values were obtained for all measurements, all of them exceeding the AUROC value of 0.93. The model obtained from the dataset of the age subgroup up to 65 years was the one that obtained the best result in all the measures, highlighting its 0.97 AUROC value. The results show the robustness of the model for all the age subgroups. Figure 3 illustrates the ROC curves corresponding to each age subgroup.

As described in Section 2.2.5, a tuning process was performed to find the optimal values for some of the hyperparameters used by the XGBoost algorithm. As can be seen in Table 3, the values obtained differ for each model, adapting to the nature of the data in each dataset. As can be seen, the simplest model, in terms of the number of trees used and their depth, is the one obtained for the over-80 subgroup.

### 3.2. Feature Selection

To evaluate the effect of the feature selection process described in Section 2.2.4, a test of the models’ performance in the process was carried out. For this purpose, we compared the AUROC and accuracy values in the intermediate model (with all variables) and in the final model (with 12 variables), using the bootstrap technique to calculate them. Table 4 shows the mean values obtained together with a confidence interval of 95%. It can be seen that performance improved in all age subgroups after performing the feature selection process. Thus, having fewer features, the models are more interpretable and, in addition, present better values for the quality measures used.

### 3.3. Feature Importance

One of the main objectives of this study is to obtain predictive models with optimal values for quality measures that are, in turn, easily interpretable by healthcare professionals. In general, the models obtained using predictive machine learning algorithms are presented as black boxes that are difficult to interpret and, therefore, it is not possible to know which variables or features have the greatest influence on the model.

**Figure 4:**
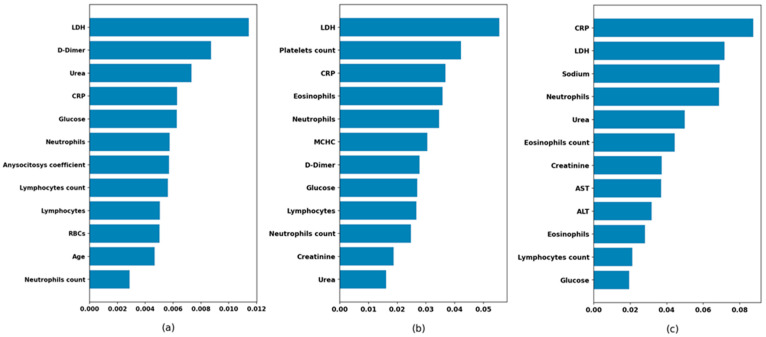
Feature importance in the final models: (**a**) up to 65 years, (**b**) between 66 and 80, (**c**) older than 80 years.

For this purpose, the present study included a process to obtain the most relevant features for the prediction of the ‘Outcome’ feature, based on the calculation of the SHAP values for each feature. Figure 4 shows the 12 features that most influence the models obtained for each age subgroup. It is noteworthy that the significance of the features, which mainly represent different laboratory measurements performed on patients, are different for each subgroup. Thus, the LDH feature is the most relevant in the (-,65] and (65,80] subgroups, and yet the most important feature in the (80,-) subgroup is the CRP. In the case of D-Dimer, it is the second most relevant feature in the (-,65] subgroup, and yet it ranks seventh in the (65,80] subgroup and is not among the 12 most relevant in the (80,-) subgroup.

## 4. Discussion

SARS-CoV-2 has produced an increase in mortality due to COVID-19. This excess mortality has been very well defined in those places where the healthcare system collapse has been more evident and has occurred much more quickly. In Italy, we have found that in the north of the country, where the pandemic began earlier, the excess mortality was much higher than in the areas of the country where there was time to establish prevention and protection measures for the population [27]. In Spain, this became evident in places with the highest population density, such as Madrid and Catalonia, as well as in regions with an older population.

The need to find an explanation for the aforementioned excess mortality has led to the stratification of the population according to different variables in order to explain higher mortality rates in each group. The first population groups that best explained the excess mortality were sex and age [28]. It is currently known that men have a higher mortality rate compared to women. This is due to different immunological mechanisms. It is true that the infection rate is the same in men and women, but later, the body’s physiological response to the virus is different depending on sex. In this sense, women display a more effective activation of the immune system. This means that women’s systems have more effective and faster clearing mechanisms for the virus than those of men. In men, the immune system undergoes a dysregulation when it comes into contact with the virus more frequently than in women, which produces a pro-inflammatory state and an increase in circulating cytokines in the blood, and as a consequence an acute inflammatory syndrome occurs with the consequent increase in mortality in this group [29]. If we add to this theory that men have a higher prevalence of cardiovascular disease, it increases the risk of mortality. If we stratify by age, it is logical to think that the older the patient, the higher the mortality, as occurs in most infectious diseases. Therefore, at an older age, mortality increased significantly [30]. Age combined with other parameters will make it possible to make a better prediction of mortality in these groups and therefore improve treatment [31]. If the risk of mortality as a function of age is divided by sex, we see that men present higher mortality than women in all age groups [32].

It is clear that stratification by age and sex is very important to understand how other factors can influence COVID-19 mortality. The individual risk factors, among which cardiovascular risk factors and diabetes stand out, will enable an approximation of the mortality risk for each individual [33].

Following the recommendations to stratify patients according to age, the proposed model includes the laboratory parameters obtained from patients upon entering the hospital. These parameters have made it possible to better understand why mortality is higher in each of the age groups, and, therefore, to carry out a treatment directed towards these alterations. In Figure 4, you can see the laboratory parameters that have been considered more clinically significant, in terms of patients who died compared to those who were discharged. As it can be seen in Table 5, all the comparisons using Mann–Whitney tests are statistically significant except in the case of creatinine in those up to 65 years of age and ALT in those over 65 years of age.

**Figure 5:**
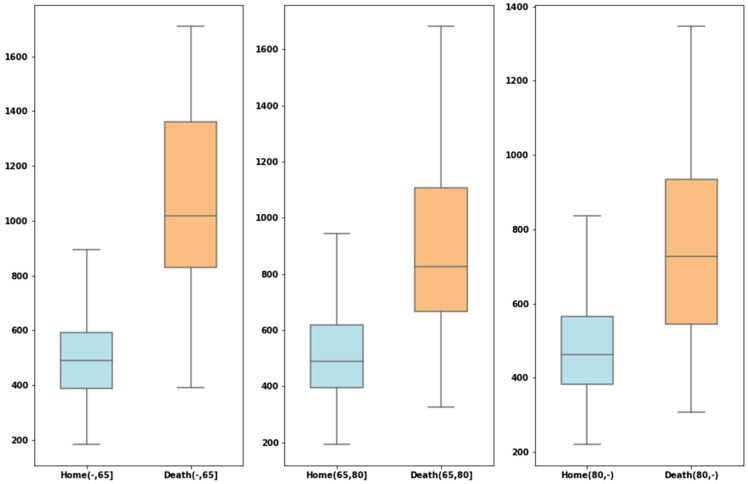
Boxplots for LDH showing the values by age subgroup based on patients who died and those who were discharged.

Figure 5 shows boxplots of LDH among patients who die and those who are discharged according to age group. It is noteworthy that in all age groups there is a clear difference between the group that dies and the one that is discharged, except in the group over 80 years of age. Both in the under-65 group and in the 65–80-year-old group, LDH is the laboratory variable that best explains mortality; however, in the group over 80 years of age, it is the second one, below CRP. It has already been established that laboratory tests better predict the severity of COVID-19 infection, with biomarkers such as D-Dimer, CRP and LDH considered as the most important in the initial screening of patients infected with COVID-19 when they visit a hospital. In this sense, the D-Dimer is the most important parameter to screen in young patients; it can even be performed in emergency units when patients are classified according to severity upon arrival [34].

**Figure 6:**
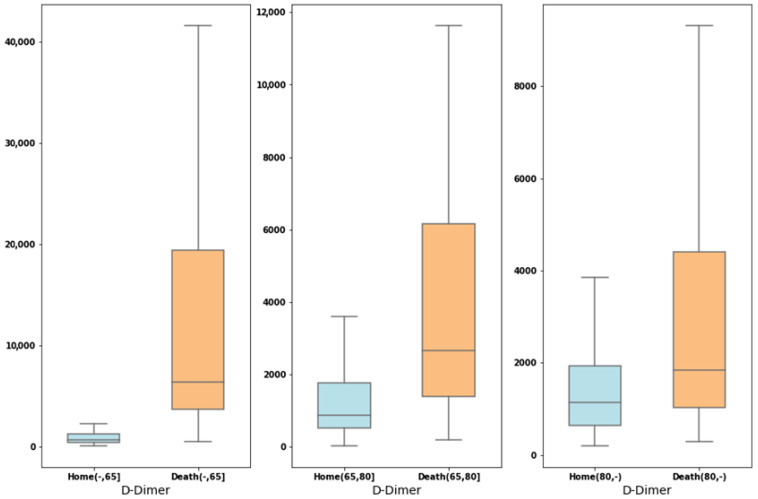
Boxplots for D-Dimer showing the values by age subgroup based on patients who have died and those who have been discharged.

**Figure A1:**
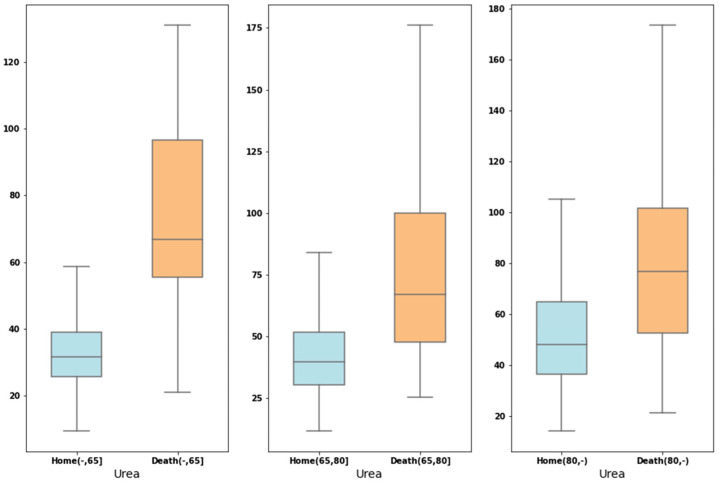
Boxplots for Urea showing the values by age subgroup based on patients who died and those who were discharged.

**Figure A2:**
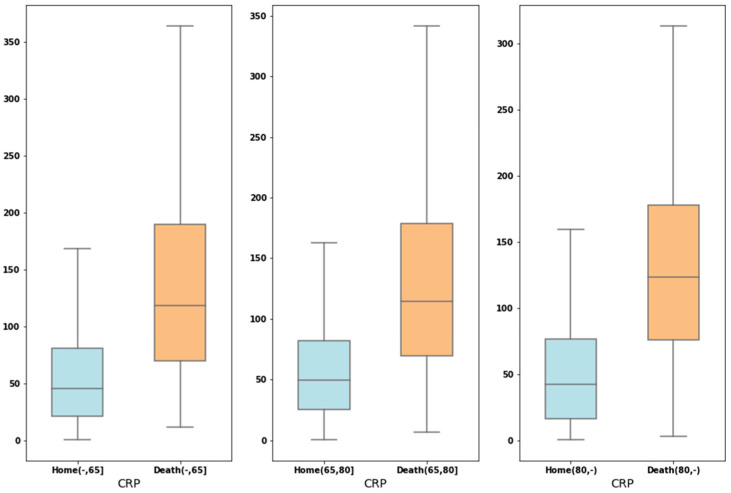
Boxplots for CRP showing the values by age subgroup based on patients who died and those who were discharged.

**Figure A3:**
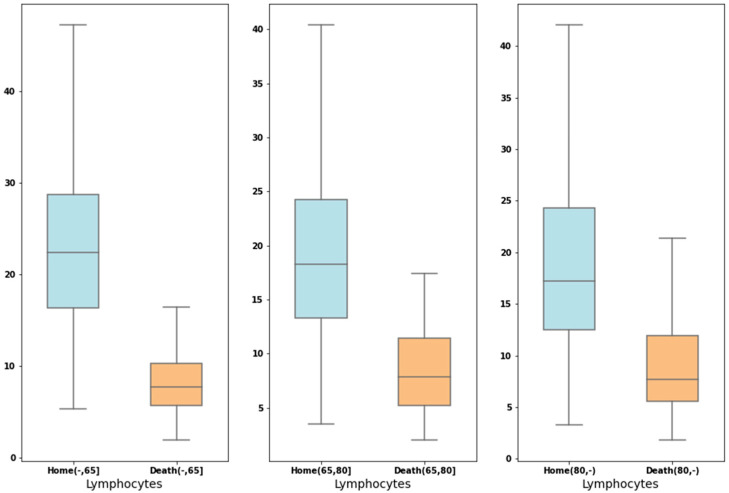
Boxplots for Lymphocytes showing the values by age subgroup based on patients who died and those who were discharged.

Figure 6 shows the corresponding boxplots for D-Dimer, and according to the results obtained using our models, Figure A1, Figure A2 and Figure A3 in Appendix A show the boxplots with significant variables regarding mortality according to age groups. In addition to LDH, the importance of CRP in the over-80 age group is noteworthy. The D-Dimer is relevant in the under-65 age group, and platelet count is important in the 65 to 80 age group. 

Furthermore, making a more exhaustive diagnosis will improve the prognosis of these patients, as the treatment will be better and more targeted, reducing the excess mortality from COVID-19 in each of the age groups. In younger patients (<65 years), establishing a treatment based on D-Dimer levels will improve the prognosis for these patients. Therefore, the benefit of establishing treatment with Enoxaparin or Apixaban will decrease mortality in those patients who have a D-Dimer greater than 1000 pg/L, compared to those patients with a D-Dimer value lower than 1000 pg/L [35]. However, in older patients, in whom inflammation measured by CRP is the best predictor of mortality, treatment should be directed towards blocking the immunological mechanisms that increase this inflammatory reaction, such as corticosteroids or IL-6 blockers (Tocilizumab or Sarilumab). The increase in therapeutic options will improve COVID-19 survival in the short term, and having successful treatments based on the risk of mortality will facilitate the work, until vaccines, in the long term, reduce mortality [36].

Having tools that allow a rapid diagnosis of COVID-19 through rapid tests such as real-time PCR in saliva [37], together with predictive models that use laboratory parameters upon arrival to the emergency room or medical center, will enable a quicker classification of patients according to their propensity to complications. Thus, patients will benefit from a more personalized treatment that will be established much earlier. All of this will translate into a reduction in COVID-19 mortality.

## 5. Conclusions

This work presents a machine learning analysis of data regarding COVID-19 outcome. Using age-stratified data, three different predictive models were trained and evaluated, mainly employing as predictor features the patients’ laboratory test results.

By employing the XGBoost method, excellent results were obtained after an evaluation using the bootstrap method on several performance metrics, showing high accuracy to predict a patient’s outcome. In addition, the importance of the features used in each of the three models was analyzed using SHAP, and differences between the three age groups were found.

## Figures and Tables

**Table 1:** Distribution of patients according to ‘Sex’ and ‘Outcome’.

		Sex		Outcome	
Dataset	Patients	Male	Female	Home	Death
Age (-,65]	798	532 (66.6%)	266 (33.3%)	769 (96.4%)	29 (3.6%)
Age (65,80]	623	377 (60.5%)	246 (39.5%)	518 (83.1%)	105 (16.9%)
Age (80,-)	402	205 (51.0%)	197 (49.0%)	274 (68.2%)	128 (31.8%)

**Table 2:** Results for the evaluation metrics in the final models.

Model	AUROC	Accuracy	Youden	Sensitivity	Specificity
Age (-,65]	0.967	0.980	0.892	0.951	0.941
Age (65,80]	0.960	0.918	0.844	0.950	0.894
Age (80,-)	0.932	0.871	0.752	0.873	0.879

**Table 3:** Values for the XGBoost hyperparameters of each model after tuning.

Hyperparameter	Age (-,65]	Age (65,80]	Age (80,-)
n_estimators	130	140	100
max_depth	5	6	2
min_child_weight	3	10	5
learning_rate	0.132	0.185	0.285
subsample	0.972	0.713	0.949
colsample_bytree	0.200	0.383	0.214

**Table 4:** Evaluation of the models before and after the feature selection process. For each metric, mean value and 95% confidence interval are shown.

	Before Feature Selection		After Feature Selection	
Model	AUROC	Accuracy	AUROC	Accuracy
Age (-,65]	0.957 (0.883–0.996)	0.978 (0.959–0.993)	0.967 (0.917–0.997)	0.980 (0.966–0.993)
Age (65,80]	0.957 (0.933–0.979)	0.916 (0.884–0.941)	0.960 (0.935–0.979)	0.918 (0.888–0.943)
Age (80,-)	0.926 (0.885–0.962)	0.867 (0.809–0.914)	0.932 (0.885–0.966)	0.871 (0.825–0.916)

**Table 5:** Results for Mann–Whitney tests (*p*-values).

Feature	Home (-,65] vs. Death (-,65]	Home (65,80] vs. Death (65,80]	Home (80,-) vs. Death (80,-)
ALT	<0.01	0.57	0.11
AST	<0.01	<0.01	<0.01
Creatinine	0.14	<0.01	<0.01
CRP	<0.01	<0.01	<0.01
D-Dimer	<0.01	<0.01	<0.01
Glucose	<0.01	<0.01	<0.01
Platelets count	<0.01	<0.01	<0.01
LDH	<0.01	<0.01	<0.01
Lymphocytes	<0.01	<0.01	<0.01
Sodium	<0.01	<0.01	<0.01
Urea	<0.01	<0.01	<0.01

## Data Availability

The data are not publicly available due to privacy reasons.

## Abbreviations

ALT	alanine aminotransferase
AST	aspartate aminotransferase
AUROC	area under the receiving operator characteristic curve
CRP	C-reactive protein
LDH	lactate dehydrogenase
MCH	mean corpuscular hemoglobin
MCHC	mean corpuscular hemoglobin concentration
MCV	mean corpuscular volume
MPV	mean platelet volume
RBCs	red blood cells
SHAP	Shapley additive explanations
XGB	XGBoost, extreme gradient boosting

## Appendix A

**Table A1:** Units and reference values for the clinical lab features in the dataset.

Feature	Units	Reference Value
ALT	U/L	<40
AST	U/L	<40
Anisocytosis coefficient	%	11.5–14.5
Basophils	%	0–1
Basophil count	1 × 10^3^/µL	0–0.1
CRP	mg/L	<5
Creatinine	mg/dL	0.6–1.0
D-Dimer	ng/mL	<500
Eosinophils	%	2–7
Eosinophil count	1 × 10^3^/µL	0.1–0.6
Glucose	mg/dL	70–105
Hematocrit	%	40–54
Hemoglobin	g/dL	13.5–17.5
LDH	U/L	120–230
Leukocyte count	1 × 10^3^/µL	4.4–11.3
Lymphocytes	%	20–48
Lymphocyte count	1 × 10^3^/µL	1.2–3.4
MCH	pg	28–33
MCHC	g/dL	33–36
MCV	fL	80–95
MPV	fL	7.4–10.4
Monocytes	%	1–11
Monocyte count	1 × 10^3^/µL	0.1–1
Neutrophils	%	40–75
Neutrophil count	1 × 10^3^/µL	1.5–7.5
Platelets count	1 × 10^3^/µL	150–450
Potassium	mmol/L	3.5–5.1
RBCs	1 × 10^3^/µL	4.1–5.9
Sodium	mmol/L	135–145
Urea	mg/dL	5–50
